# Graphene-Based Biosensors with High Sensitivity for Detection of Ovarian Cancer Cells

**DOI:** 10.3390/molecules26237265

**Published:** 2021-11-30

**Authors:** Qi Wan, Ling Han, Yunchuan Guo, Huijun Yu, Li Tan, Ai Zheng, Yali Chen

**Affiliations:** 1 Department of Gynecology and Obstetrics, West China Second Hospital, Sichuan University, Chengdu 610041, China; wanqi123@163.com (Q.W.); hanlingluobo@sina.com (L.H.); 2 Key Laboratory of Birth Defects and Related Diseases of Women and Children (Sichuan University), Ministry of Education, Chengdu 610041, China; 3 Department of Reproductive Medicine, Chengdu Jinjiang Maternal and Child Health Hospital, Chengdu 610041, China; yuhj@jxr-fertility.com (H.Y.); tanl@jxr-fertility.com (L.T.); 4 Chengdu Ginkgo Electronics Technology Co., Ltd., Chengdu 610213, China; y.guo@ginkgoet.cn

**Keywords:** graphene-based biosensor, circulating tumor cell (CTC), ovarian cancer

## Abstract

Ovarian cancer has the highest mortality rate in the world. Therefore, it is urgent but still challenging to develop an efficient circulating tumor cell (CTC) detection method to sensitively detect ovarian cancer. To address such issues, herein, for the first time, we present a novel CTC detection method for ovarian cancer cells by designing sensitive and rapid graphene-based biosensors. This graphene-based sensor, consisting of a cell pool and two electrodes, can be prepared by a conventional chip fabrication process. It demonstrates high-sensitivity detection even for several ovarian cancer cells by comparing the electrical signal before and after adding cell solution. Moreover, the graphene-based biosensors can perform rapid detection with good repeatability. This suggests that this novel method is possible to use for the early detection of ovarian cancer with very low CTC cell concentration. This work provides a novel and quick strategy to detect ovarian cancer and further judge or predict the risk of the transfer of ovarian cancer.

## 1. Introduction

Ovarian cancer has the highest mortality rate in the world. The main reason for the high mortality rate is that about 75% of patients are in an advanced stage when they consult for medical advice, and only 25% of patients are found in the early stage [1]. Metastasis is the main cause of death in cancer patients. Solid tumors such as ovarian cancer, when the lesions can be found by conventional detection methods such as ultrasound examination or radiological examination, are often in an advanced stage, with poor treatment effect and high mortality. In total, 70% of patients are diagnosed when the cancer is in an advanced stage or with multiple site metastasis, with 55~75% of the patients suffering remission after clinical first-line treatment relapsed within 2 years [2], and the 10 year disease-free survival rate of patients with recurrence was less than 15% [3]. Therefore, timely detection and treatment before cancer metastasis is the most important factor related to the prognosis of patients.

The existing screening programs mainly rely on ultrasound and serum tumor markers, but the specificity and sensitivity of these methods are not high enough [4]. In recent years, the detection and research of circulating tumor cells (CTCs) has gradually become the focus of tumor research. CTCs refer to tumor cells released into peripheral blood with blood circulation after primary tumor or metastatic tumor shedding. Theoretically, the occurrence of CTCs in peripheral blood is the premise of tumor distant metastasis and the key link in the formation of metastatic tumors [5]. CTCs are considered as seed cells of metastasis. The number of blood cells per milliliter of blood is more than 10^9^, while the number of CTCs is only a few to hundreds. The relationship between the number of CTCs and the therapeutic effect on patients has been investigated in a considerable number of studies. The results of Tewes et al. [6] showed that the number of CTCs increased continuously before and during treatment, which often indicated a shorter survival period, while Satelli et al. [7] combined the detection of epithelial cell adhesion molecule (EpCAM) and mesenchymal and epithelial–mesenchymal transformed (EMT) cells to isolate CTCs, and it was found that there were significant differences in the number of CTCs between the two groups. At present, the cancer curative efficacy evaluation is based on imaging data. However, the delay of imaging data reflecting the disease may lead to some patients receiving ineffective treatment, thus delaying the time for patients to choose effective treatment drugs. The research results of Budd et al. [8] showed that CTC count may reflect the treatment effect earlier than imaging, and some patients may receive radiotherapy or have a local inflammatory reaction. Patients may have an abnormal uptake of 18f-fdp, which may lead to false positive results with Positron Emission Tomography-Computed Tomography (PET-CT). Therefore, from this point of view, the CTC count may be better than PET-CT for judging the therapeutic effect of patients.

In recent years, many studies have confirmed that CTCs in peripheral blood are closely related to tumor metastasis [9,10,11]. With the gradual progress of detection technology, the significance of CTCs in the diagnosis, treatment effect and prognosis of patients with malignant tumor has attracted increased attention. At present, CTC detection technologies mainly include physical methods, such as CTC size, density and charge, and chemical methods based on antigen–antibody reaction, such as the immunomagnetic bead method [9], microfluidic chip method [10], density gradient centrifugation method [11], etc. The advantages of the physical enrichment method are its simple operation, relatively low cost, little impact on the activity of CTCs and convenient downstream analysis, avoiding the omission of CTCs with specific immune markers to make the types of CTCs more comprehensive; however, there are still some blood cells with similar physical characteristics as CTCs, which will lead to the low purity of CTCs and the difficulty of counting. In the separation process based on antigen–antibody reaction, CTCs that do not express markers such as EpCAM and cytokeratin (CK), such as stromal type and epithelial stromal type, will be missed. At the same time, they will be mixed with normal circulating epithelial cells, leading to false negative and false positive results. Therefore, it is necessary to explore new highly sensitive detection methods.

Graphene is a two-dimensional (2D), single-atom-thick semiconductor with sp^2^-bondedcarbon atoms arranged in a honeycomb lattice. Due to its 2D honeycomb-like crystalline structure and linear dispersive band structure, graphene has many potential applications in microelectronics, optoelectronics, biomedical sensing, etc. Graphene-based biosensors have been intensively investigated in enzymatic bio-sensing [12], DNA sensing, immune-sensing [13] and cancer cell-sensing [14,15]. A suspended single crystalline graphene (SCG) biosensor has been reported for the detection of multiplex lung cancer tumor markers [16]. Peng et al. [17] utilized graphene quantum dots to visualize human breast cancer cell T47D. However, there are still no reports about the use of graphene to effectively detect the ovarian cancer cells.

Herein, we present a novel graphene-based ovarian cancer sensor consisting of a cell pool and two Au electrodes. The graphene sensor demonstrates high sensitivity to detect several ovarian cancer cells by comparing the electric signal before and after adding cell solution, based on the resistance change caused by the adsorption of ovarian cancer cells on the graphene surface. It can judge or predict the risk of the transfer of ovarian cancer. This work presents a novel, facile and quick method to detect ovarian cancer with high sensitivity.

## 2. Materials and Methods

### 2.1. Growth and Transfer of Graphene Film

In this work, the graphene film was first grown on the surface of Cu foil (Alfa Aesar, No. 13382) by chemical vapor deposition (CVD) [18]. Then, PMMA was spin-coated on the surface of graphene/Cu foil, forming a PMMA/graphene/Cu sandwich like structure. Subsequently, the underlying Cu foil was etched with 1M FeCl_3_ solution. The PMMA/graphene was cleaned in DI water for 30 min and then transferred onto the SiO_2_/Si substrate. Finally, the PMMA was removed by acetone.

### 2.2. Fabrication of Graphene-Based Biosensors

The graphene-based senor was simply fabricated on graphene/SiO_2_/Si via a conventional chip fabrication process. Firstly, 1 cm × 1 cm square graphene patterns were obtained via photolithography and oxygen plasma etching. Then, Au electrodes with a thickness of 50 nm were fabricated by depositing Au followed by a lift-off process. Finally, a cell pool in the center of each graphene pattern was constructed by using silicone gel. A graphene-based biosensor on SiO_2_/Si substrate with graphene, Au electrode and cell pool was obtained.

### 2.3. Culture of SKOV3 Ovarian Cancer Cells

SKOV3 ovarian cancer cell series (provided by the public laboratory of the Second Affiliated Hospital of West China) were cultured in RPMI-1640 (Transgene, Illkirch Graffenstaden, France) complete medium containing 10% calf serum (Gibco, Frederick, MD, USA) under the condition of 5% CO_2_ and 37 °C.

### 2.4. Preparation of Cancer Cell Solution and Electrical Test

The solutions with various cancer cell numbers were prepared as follows. The SKOV3 cells were inoculated into the six-well plate with 5 × 10^4^ cells/well. The cells to be tested were discarded from the culture medium and washed with PBS three times. The cells were gently blown, and the cell suspension was collected to prepare a cell suspension with a concentration from ca. 200 to 200,000 cells μL^−1^. The solution without or with various cancer cells was investigated by testing the electrical signal change before and after adding the solution into the cell pool. Firstly, before we added the solution into the cell pool, we applied a fixed voltage (e.g., 0.01 V) and started to record the current time data; during the test process, we added 50 μL of solution dropwise with a pipette, and we could observe that the current signal obviously changed. By comparing the signal just before and after adding the solution without and with various cell numbers, we were able to obtain useful information.

## 3. Results and Discussion

### 3.1. Morphology and Structure of Graphene Film and Photos of Graphene-Based Biosensor

**Figure 1:**
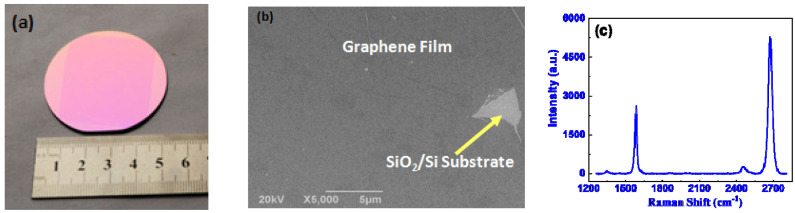
The photo of large-area graphene film transferred onto 2 inch SiO_2_/Si substrate (**a**), SEM image (**b**) and the Raman spectrum of the graphene film (**b**).

The large-area graphene film grown on Cu foil by CVD was transferred onto 2 inch SiO_2_/Si substrate by a standard PMMM-assisted transfer process. The photo of large-area graphene film transferred onto 2 inch SiO_2_/Si substrate is shown in Figure 1a. One can see that the transferred film is very homogeneous, and nearly no defects can be observed with the naked eye. The morphology of graphene transferred onto SiO_2_/Si substrate is further characterized by a scanning electron microscope (SEM). As shown in Figure 1b, one can see that the graphene film is homogeneous except for the damaged area. The Raman spectra of such transferred samples are measured. As shown in Figure 1b, the G-to-2D intensity ratio (*I*_G_/*I*_2D_) of 0.49 indicates that the graphene film transferred on SiO_2_/Si substrate is monolayer, and the D-to-G intensity ratio (*I*_D_/*I*_G_) is as low as 0.038, which suggests that the graphene film has high quality [19,20,21,22].

**Figure 2:**
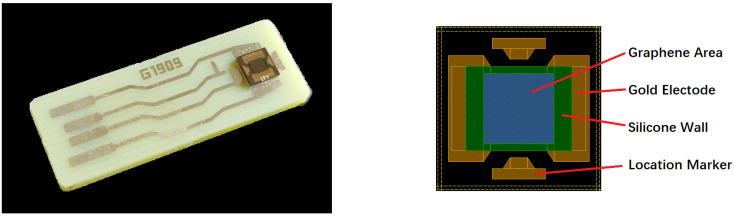
The photo of the GBC-100 graphene-based biosensor.

The graphene/SiO_2_/Si substrate was cut into many ~2 × 2 cm^2^ pieces and packaged as GBC-100 graphene-based biosensors by Chengdu Ginkgo Electronics Technology Co., Ltd. The photo of the GBC-100 biosensor is shown in Figure 2.

### 3.2. Biosensing Properties

**Figure 3:**
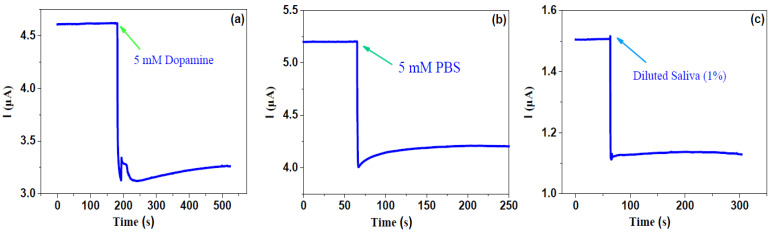
Response for 5 mM PBS, 5 mM dopamine and 1% diluted saliva solution.

Firstly, the electrical response for the 5 mM phosphate buffer saline, 5 mM dopamine and diluted saliva (1%) without any cancer cells were measured. The time dependence of the current response for such liquids was recorded at a fixed voltage of 0.01 V before and after putting them into the cell pool. As shown in Figure 3, one can observe that before dipping the liquid, the current remained constant; when such liquids were put into the cell pool, the current quickly decreased and then slowly maintained a new balance. The response is defined as *η* = (*I*_0_ − *I*)/*I*_0_ × 100%, where *I*_0_ is the current just before dipping the liquid and *I* is maximal (or minimal) value after dipping the liquid, which is similar to the optoelectronic response of 2D semiconductor detectors or sensors [23,24,25]. One can see that the responses for phosphate buffer saline, dopamine and diluted saliva are 19.23%, 32.10%, 25.17%, respectively. This suggests that the graphene-based biosensor is very sensitive for even reference liquids. In addition, it indicates that after dipping such liquids, the resistance of graphene increases.

**Figure 4:**
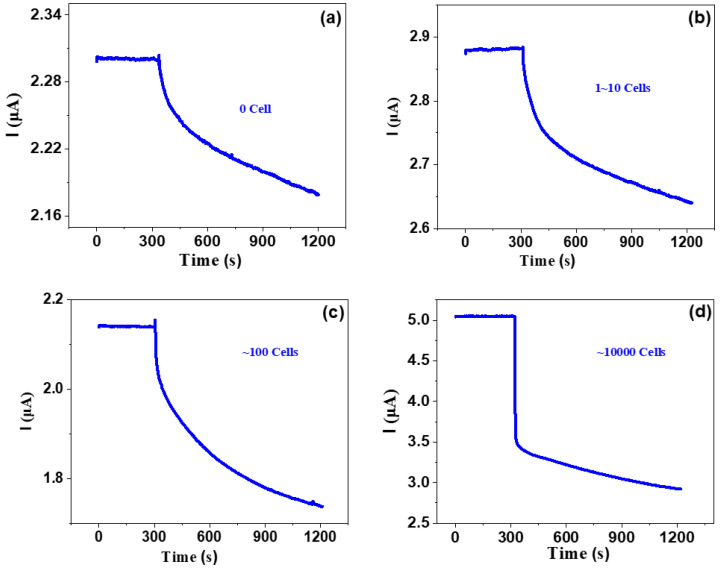
Response of various concentrations of ovarian cancer cells.

Then, we investigate the response for various concentrations of ovarian cancer cells, as shown in Figure 4. One can see that all curves show similar trends; that is, just after dipping the solution without or with various cancer cells, the current quickly decreased and then gradually decreased, with current-decrease trends more or less similar to those of phosphate buffer saline, dopamine and diluted saliva. However, the changing rates are very different from those of phosphate buffer saline, dopamine and diluted saliva. The responses of the bare solvent and the solution with 1~10, ~100 and ~10,000 cancer cells are 3.5%, 5.6%, 16.8% and 33.3%, respectively. This indicates that as the cancer number increases, the response significantly increases. It is noted that even several cancer cells can be definitely detected, which suggests that our graphene-based biosensor has very high detection sensitivity. This work presents a very efficient way to detect blood with low-concentration cancer cells, which suggests that the graphene-based biosensor is very suitable for detecting CTC-based cancer cells.

**Figure 5:**
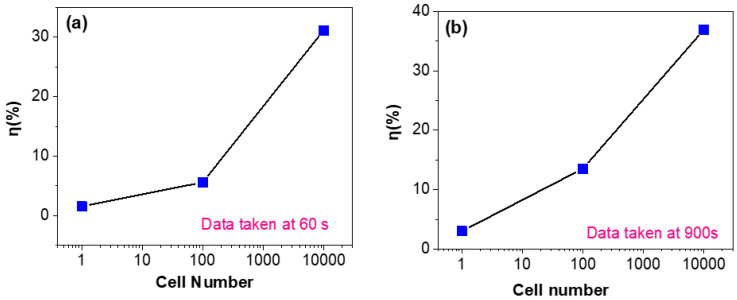
Cell number dependence of sensitivity after a fixed detection time of 60 s (**a**) or 900 s (**b**).

The response sensitivity with cell number at various fixed detection times of 60 and 900 s is investigated. The typical data are shown in Figure 5. As shown in Figure 5a, when the detection time is fixed at 60 s, one can observe that as the cell number increases from several to ten thousand, the response sensitivity increases from 2% to ~30%. As shown in Figure 5b, when the detection time is fixed at 900 s, the response sensitivity increases from 3% to ~37%. From these results, we can make two important conclusions: (i) although the detection time is as short as 1 min and the cancer cell number is as low as ~1 (the early stage in CTCs), the response sensitivity is high enough, which suggests that the present detection method based on a graphene-based biosensor is very sensitive and rapid; (ii) if the response sensitivity for some cancer cells is not very high, one can improve it by simply increasing the detection time.

**Figure 6:**
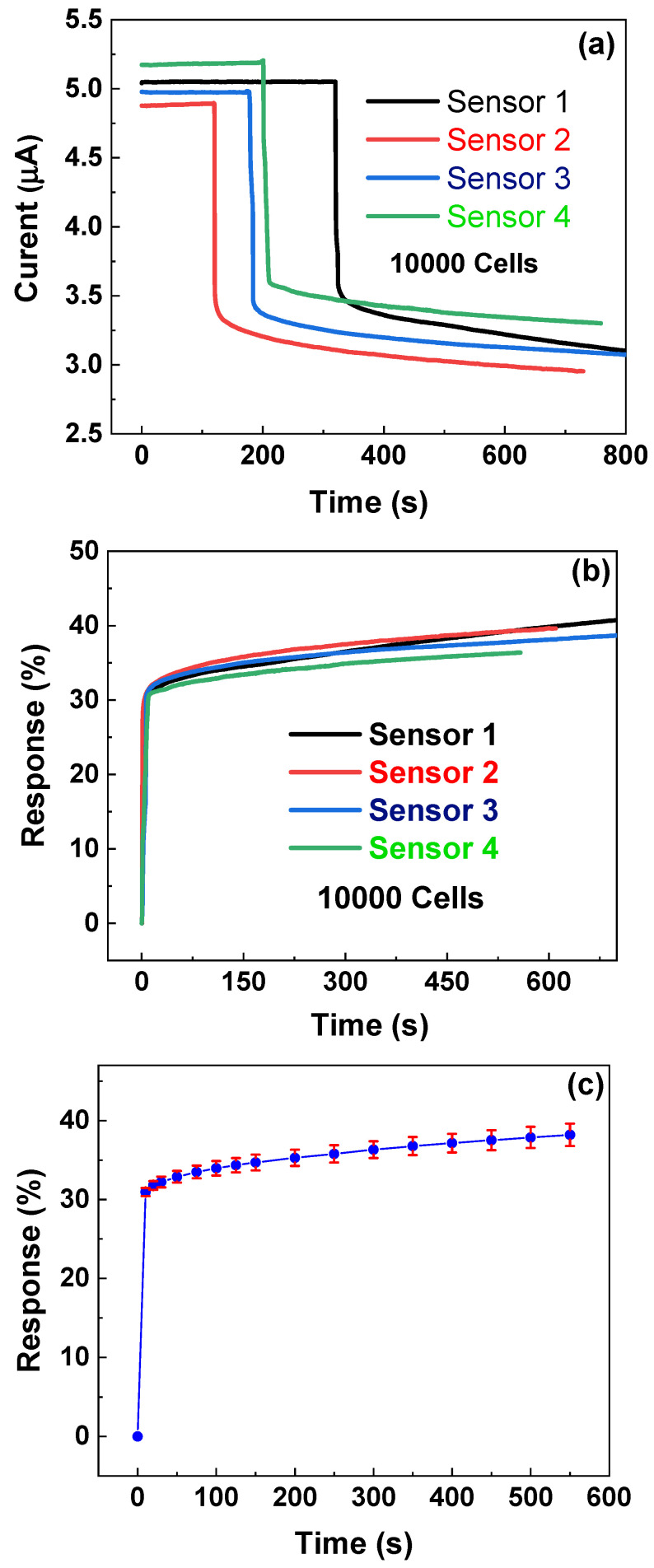
Time dependence of currents (**a**) and response (**b**) of four different sensors. (**c**) Time dependency of response with error bars; data calculated from (**b**).

The time dependence of the response for four different biosensors at a fixed cancer number of 10,000 is investigated. As shown in Figure 6a, one can observe that that although the i~t currents are very different for four biosensors, the change trends for four biosensors after dipping CTC solutions are very similar: before dipping solutions, the currents for all four biosensors nearly remain unchanged as time increases, and after dipping solutions, the currents of all four biosensors sharply decrease with time and then slowly decrease with increasing time. It is noted that at a fixed voltage of 0.1 V, four biosensors show somewhat different currents before dipping cell solutions, which might be caused by the different PMMA residues in different zones during transfer and the different contact resistance of metal electrodes. This can be more or less resolved in future by using a better transfer process and more strict photolithography and metal deposition process. In order to allow a better comparison between the four biosensors, we calculate the corresponding response *η* = (*I*_0_ − *I*_t_)/*I*_0_ × 100%, where *I*_0_ is the current just before dipping the liquid, and *I*_t_ is the current value after dipping the liquid for t seconds. As shown in Figure 6b, one can observe that all four different sensors demonstrate very similar change trends of *η* ~ t. Moreover, the responses for all four sensors reach a high response over 30% within 10 s, which suggests the graphene-based biosensors can rapidly detect the ovarian cancer cells.

In order to further investigate the repeatability of the responses of four different biosensors, according to Figure 6b, we calculate the mean and standard deviation and plot *η* ~ t with an error bar. As shown in Figure 6c, one can observe that with a 600 s measurement period, the *η* ~ t curve shows small error bars with a maximal (standard deviation/mean) ratio of 3.7%, which suggests that graphene-based biosensors have good repeatability for detecting ovarian cancer cells.

As mentioned above, this work provides a very effective method for the detection of low concentrations of cancer cells in blood, which shows that the graphene-based biosensor is promising for the detection of tracing cancer cells in blood.

## 4. Conclusions

CTC detection is expected to be a potential method for the early detection of ovarian cancer. It is of great significance for the early diagnosis and treatment of ovarian cancer to detect CTCs in patients’ blood with high sensitivity. Herein, we have fabricated a graphene electrode by a relatively simple method. This kind of biosensor based on graphene is even very sensitive to the reference liquid. We can compare the CTCs in the blood of tumor patients with different reference liquids to find even a very small number of CTCs in the blood.

Using the electrical response of different concentrations of ovarian cancer cells, we found that the current changing trend of the ovarian cancer cell suspension was similar to that of phosphate buffered saline, dopamine and diluted saliva. The graphene-based biosensors show good repeatability. With the increase of the number of cancer cells, the response changes significantly, but even a few cancer cells can be detected, which shows that our graphene-based biosensor has very high detection sensitivity. In this way, we can detect a very small number of CTCs in the blood to detect early ovarian cancer and predict tumor metastasis.

## Data Availability

Authors can confirm that all relevant data are included in the article.
